# Supplementary figures and images for: Branch Mode Selection during Early Lung Development
Source: PLoS Comput Biol. 2012 Feb 16;8(2):e1002377. doi: 10.1371/journal.pcbi.1002377 (PMC3280966; doi:10.1371/journal.pcbi.1002377)

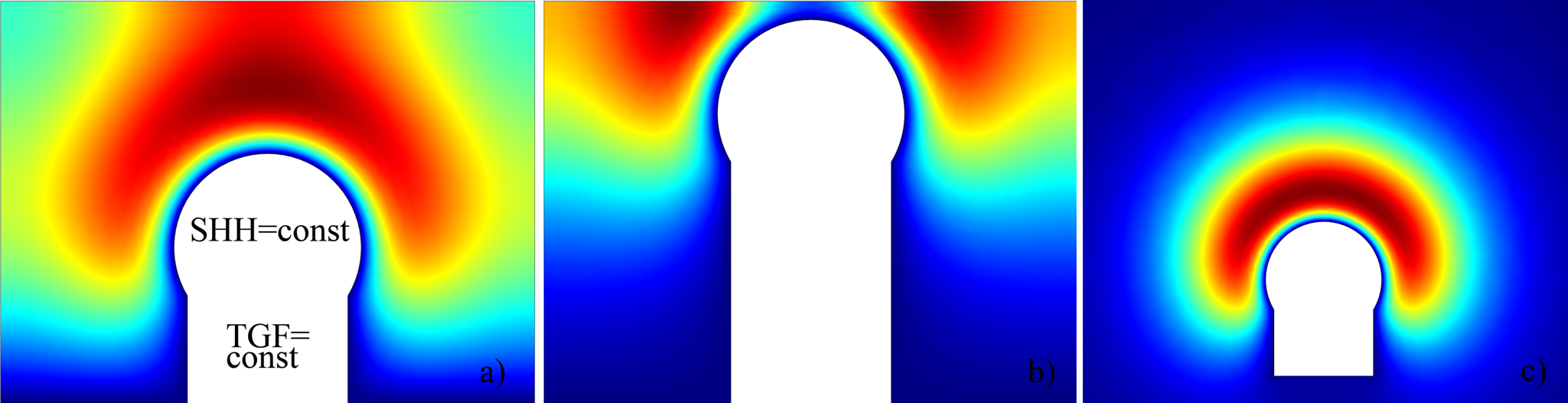

Supplement: Figure S1 — FGF distribution at the steady state calculated according to the model presented by Hirashima et al [44] . SHH concentration is fixed at the lung tip and FGF production is promoted by SHH. The computational domain is equal to that shown on sub-figures a) and b). a) Elongation mode is observed when lung tip is far from the impermeable domain boundary, b) planar bifurcation is observed when the lung tip is in the proximity of the impermeable domain boundary. c) FGF expression pattern calculated on the infinitely big domain, in this case FGF distribution is always corresponds to elongation mode. Note, that panel c) is scaled differently compared to a) and b); stalk and tip radius are the same in all panels. (TIF) [file pcbi.1002377.s001.tif]

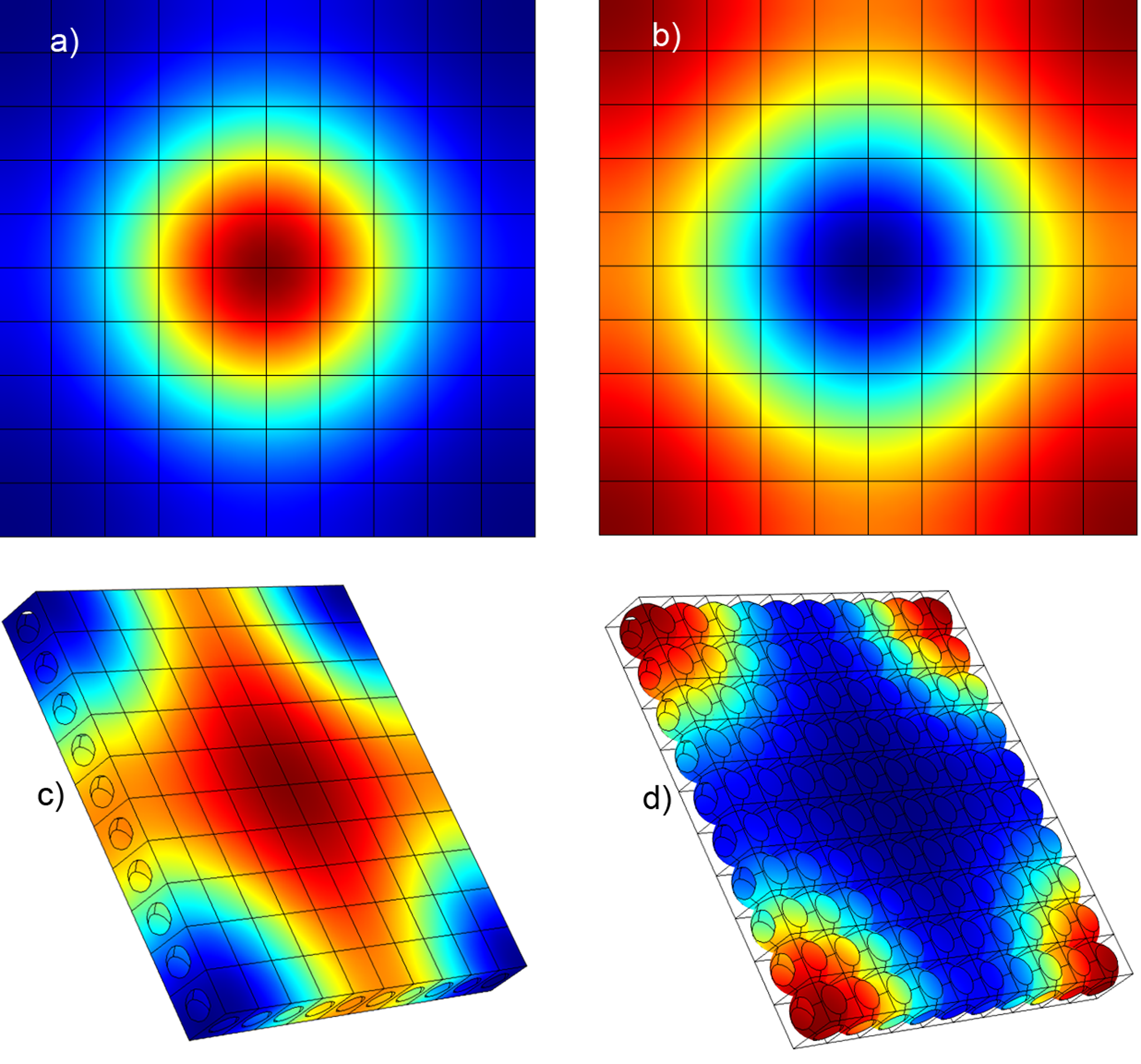

Supplement: Figure S2 — Turing pattern on the continuos domain and on the array of cells. The steady state distribution of the fast diffusion component a), c) and slowly diffusion component b), d) in a Schnakenberg model. Upper and lower rows show the solution of the Schnakenberg model on 2D plane and on an array of spheres, correspondingly. (TIF) [file pcbi.1002377.s002.tif]

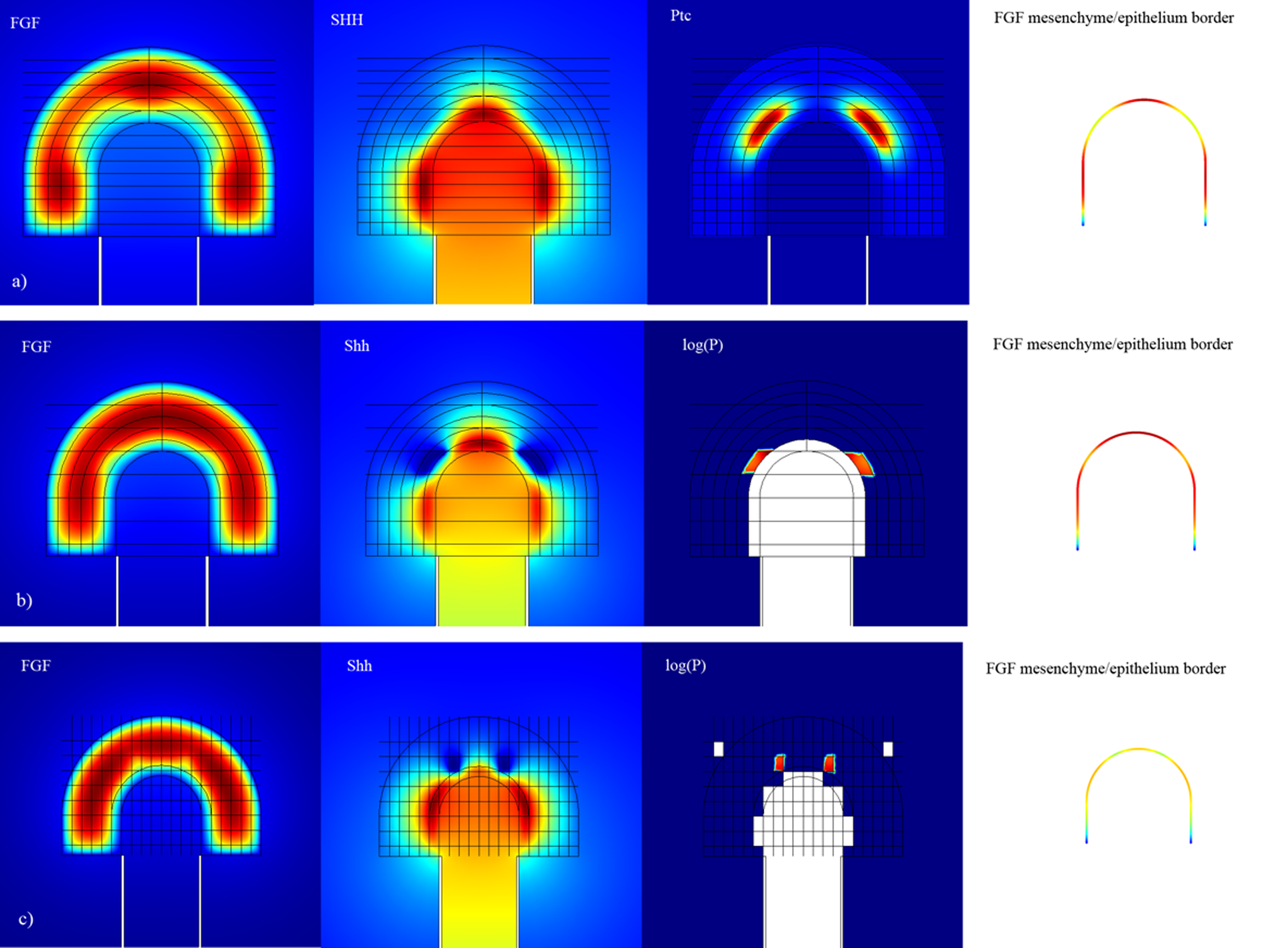

Supplement: Figure S3 — The steady state distributions of the concentrations of FGF10, SHH and the receptor Ptc on a domain that is divided into cells. a) If the diffusion coefficient of Ptc at the cell edge is significantly lower than on the cell” surface ( = 0.001) then the observed patterns are the same as in a continues model (compare to Figure 2a). b, c) The diffusion coefficient of Ptc is set to zero at the cell” edge (black lines on the domain), panels b) and c) show pattern of the domain split into deformed rectangular and rectangular “cells”, correspondingly. In this case the observed patterns are distorted from those observed in the case of the continuous model (Figure 2); however, all main features are preserved, in particular at the mesenchyme/epithelium border (right column). Unless stated otherwise the parameter values in Table 1 were used. (TIF) [file pcbi.1002377.s003.tif]

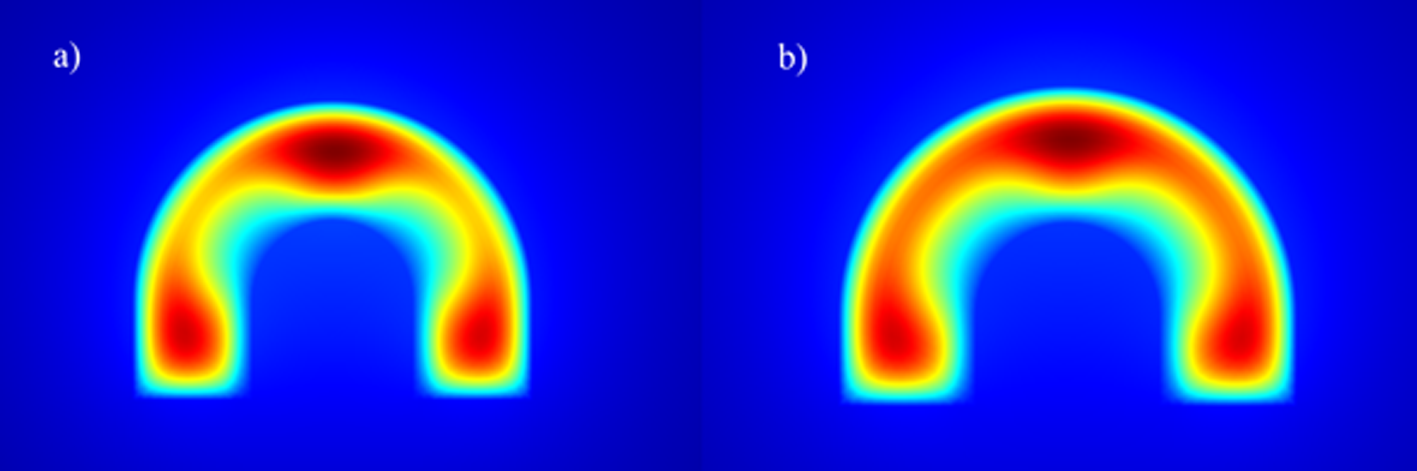

Supplement: Figure S4 — The steady state distribution of FGF10. a) n = 1, b) n = 3 parameters of other values are similar to that presented in Table 1 . (TIF) [file pcbi.1002377.s004.tif]

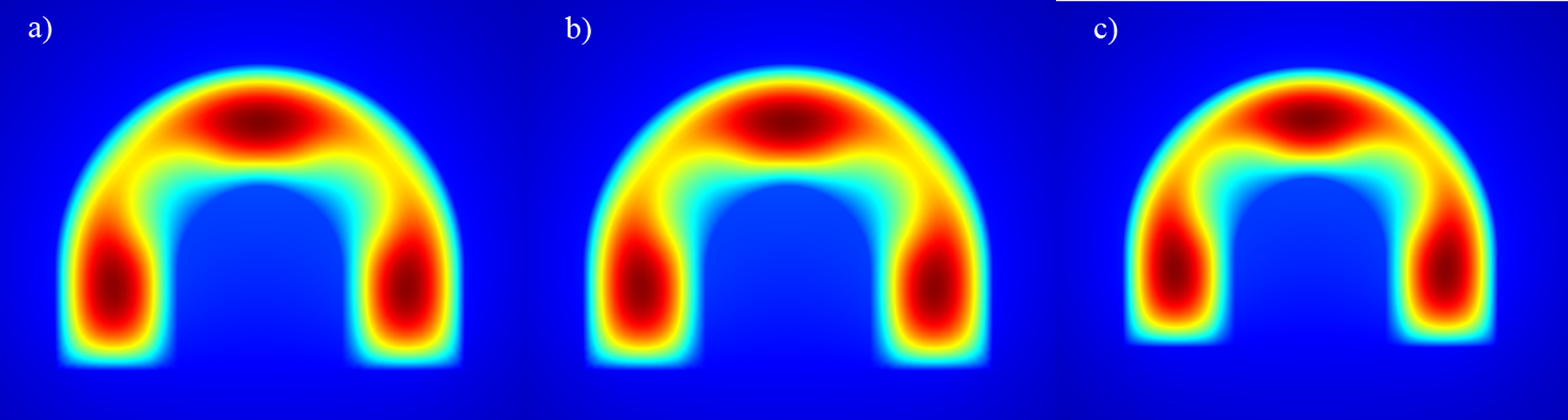

Supplement: Figure S5 — The steady state distribution of FGF10 calculated assuming various SHH-Ptc complex stoichiometry a) , b) , c) . The values of the other parameters are similar to the ones in Table 1, except for case c) where Dp = 0.004. (TIF) [file pcbi.1002377.s005.tif]

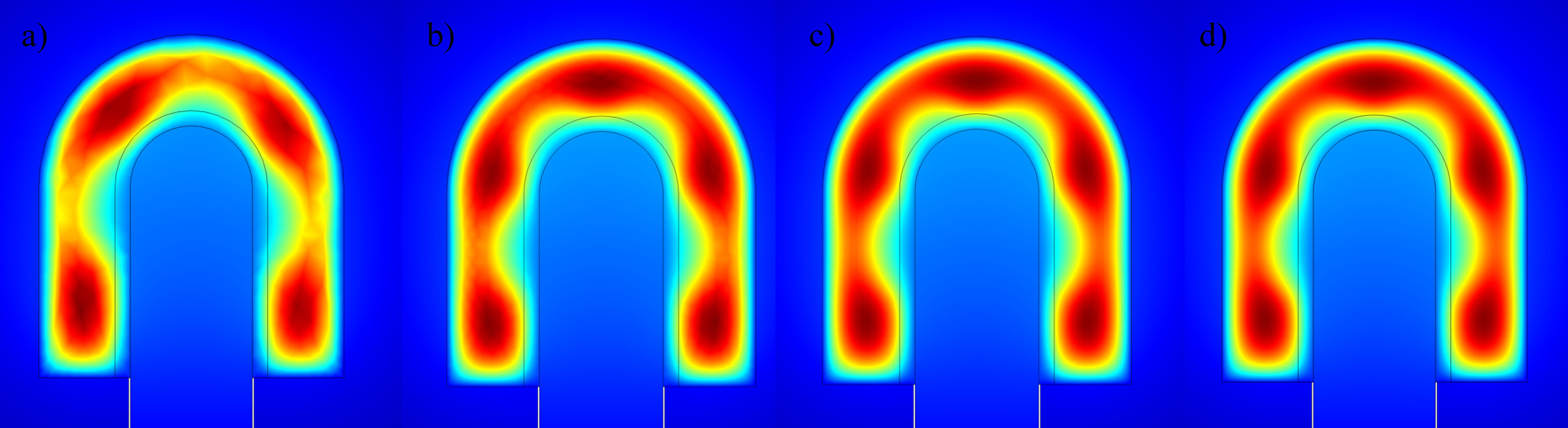

Supplement: Figure S6 — The impact of mesh resolution. FGF distribution at calculated on a mesh with a maximum element size of a) 0.4, b) 0.2, c) 0.1 and d) 0.05. (TIF) [file pcbi.1002377.s006.tif]

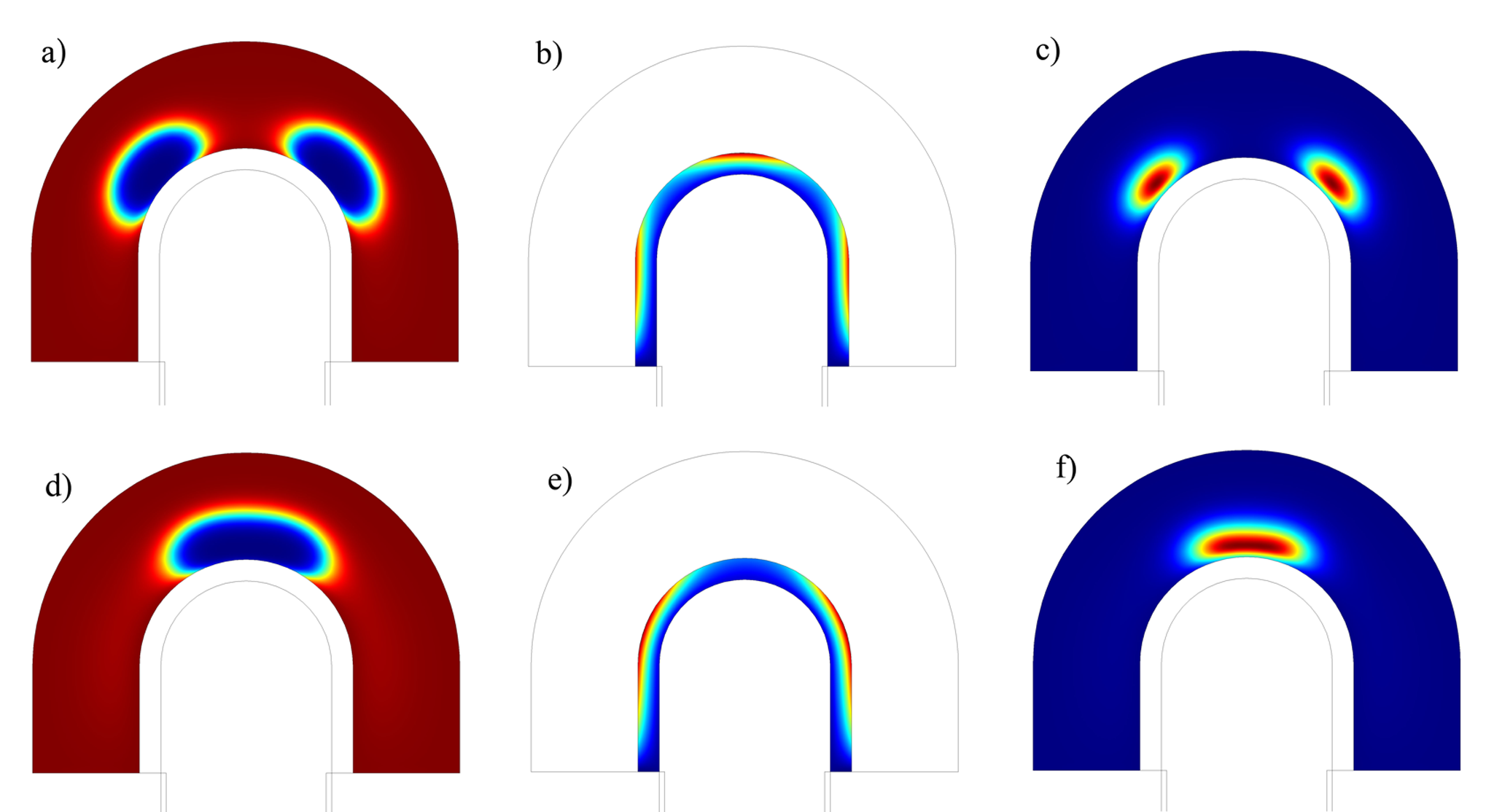

Supplement: Figure S7 — SHH, FGF and receptor Ptc expression patterns at the steady state. Expression patterns of (a,d) FGF10, (b,e) SHH, and (c,f) Ptc in the steady state for parameter values as in Table 1 (a–c) or with (d–f). The upper panel presents an example of FGF10 distribution during the lateral branching mode, while the lower panel provides an example for FGF10 distribution during a bifurcation branching mode. (TIF) [file pcbi.1002377.s007.tif]

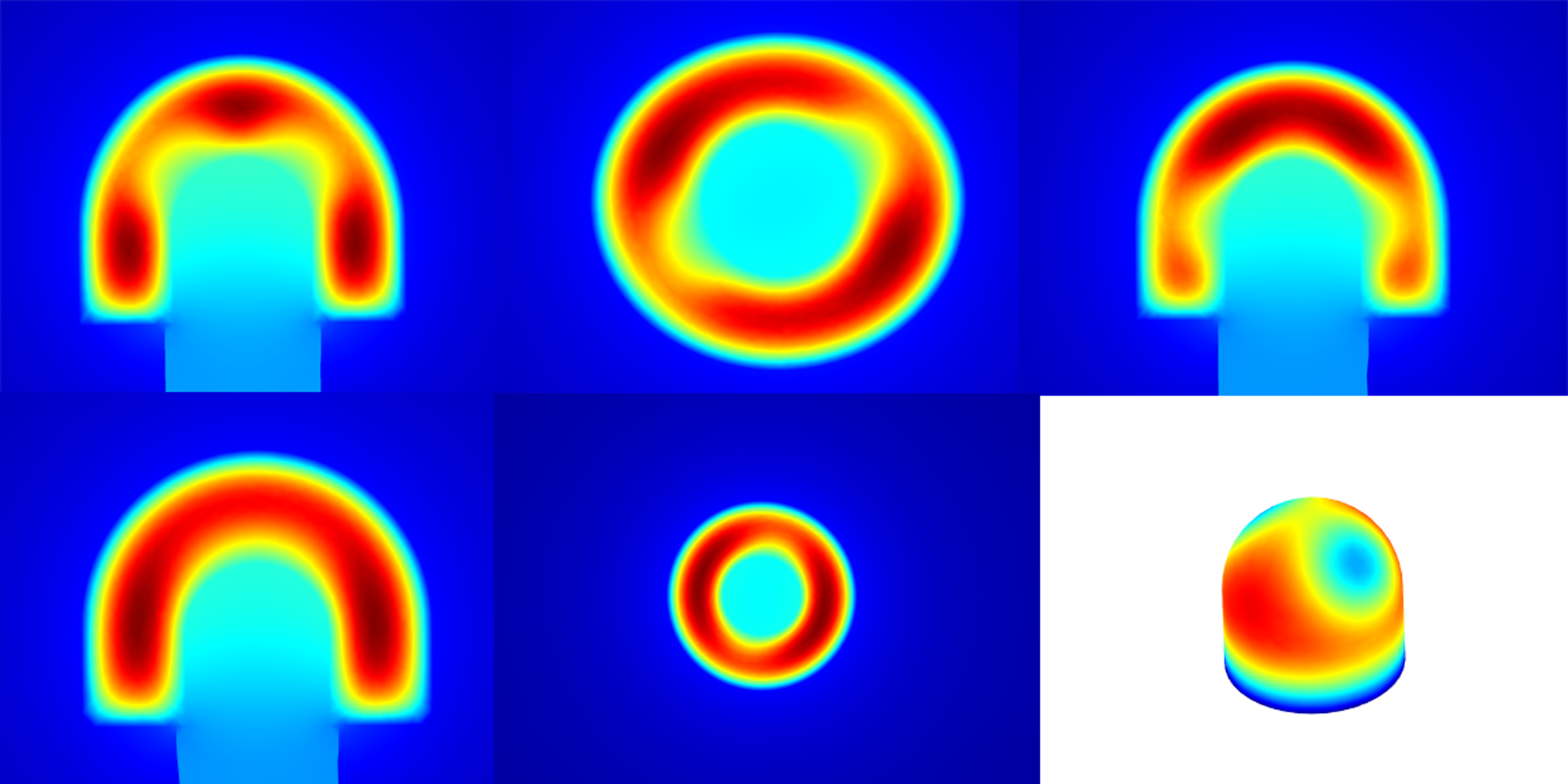

Supplement: Figure S8 — FGF pattern in 3D. The steady state distribution of FGF10 in 3D. Upper and lower panel show lateral branching and bifurcation modes of branching, correspondingly. Parameter values are as similar to those indicated in Table 1. (TIF) [file pcbi.1002377.s008.tif]

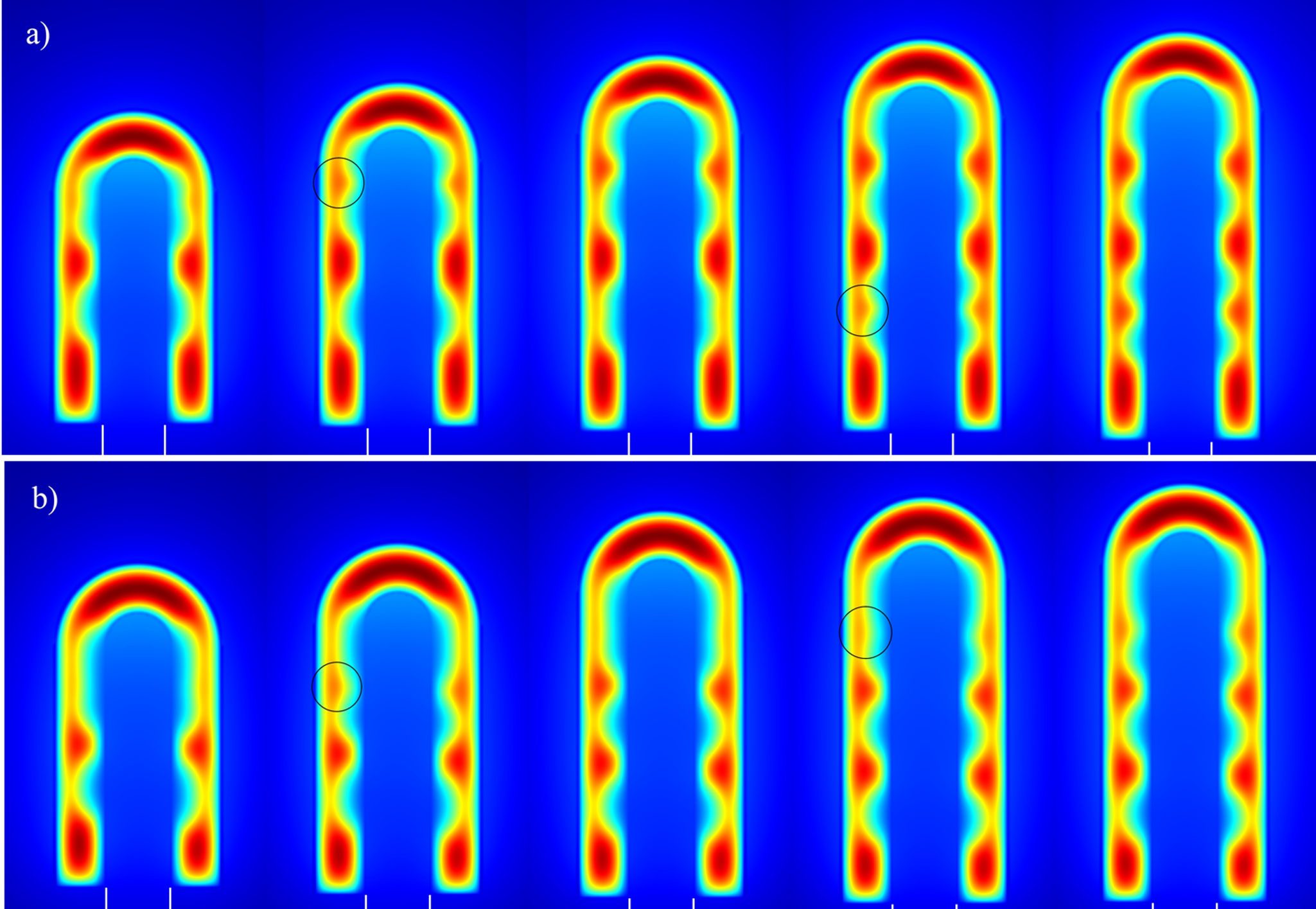

Supplement: Figure S9 — The impact of growth mode on a pattern. The FGF distribution on a growing lung: uniform growth a), local growth at the tip b).Parameters values used to simulate domain growth in the local growth mode are equal to that given in Table 1, except initial stalk length and in the case of domain stretching , , , , , and the rest of parameters are as given in Table 1. (TIF) [file pcbi.1002377.s009.tif]

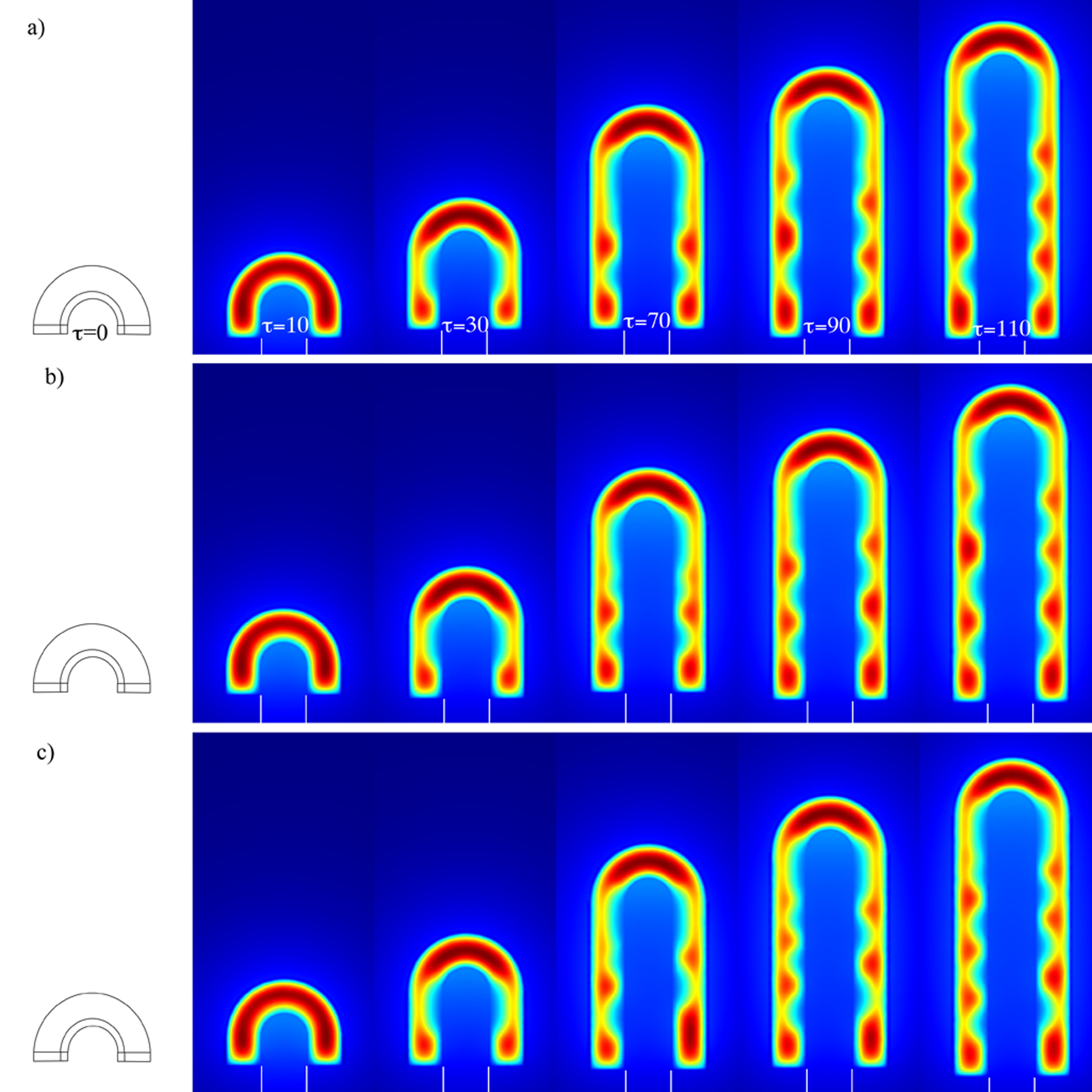

Supplement: Figure S10 — The robustness of FGF pattern to parameter variability. FGF pattern calculated on a growing domain with parameter distributed normally with standard deviation equal to 0.1 a), 0.2 b) and 0.3 c) of the corresponding value given in Table 1. (TIF) [file pcbi.1002377.s010.tif]

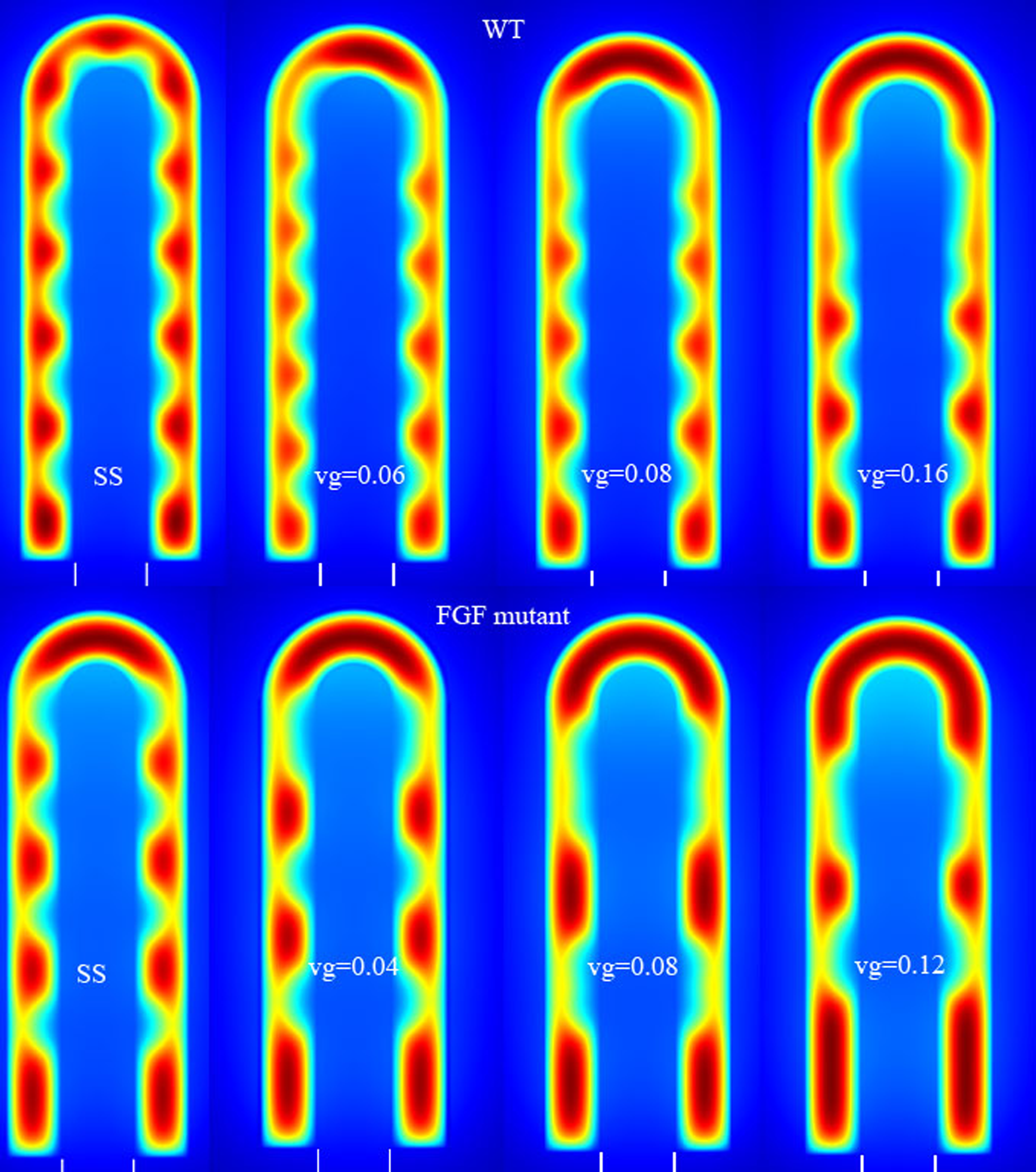

Supplement: Figure S11 — Dependence of FGF pattern in the wild type and FGF mutant lung on the lung growth rate. The FGF pattern were simulated for (top row) wildtype conditions (Table 1) and (bottom row) mutants with lower FGF expression ( = 2.6) on constant (SS) and growing domains (growth speed ). Pattern are compared on equally sized domains. (TIF) [file pcbi.1002377.s011.tif]

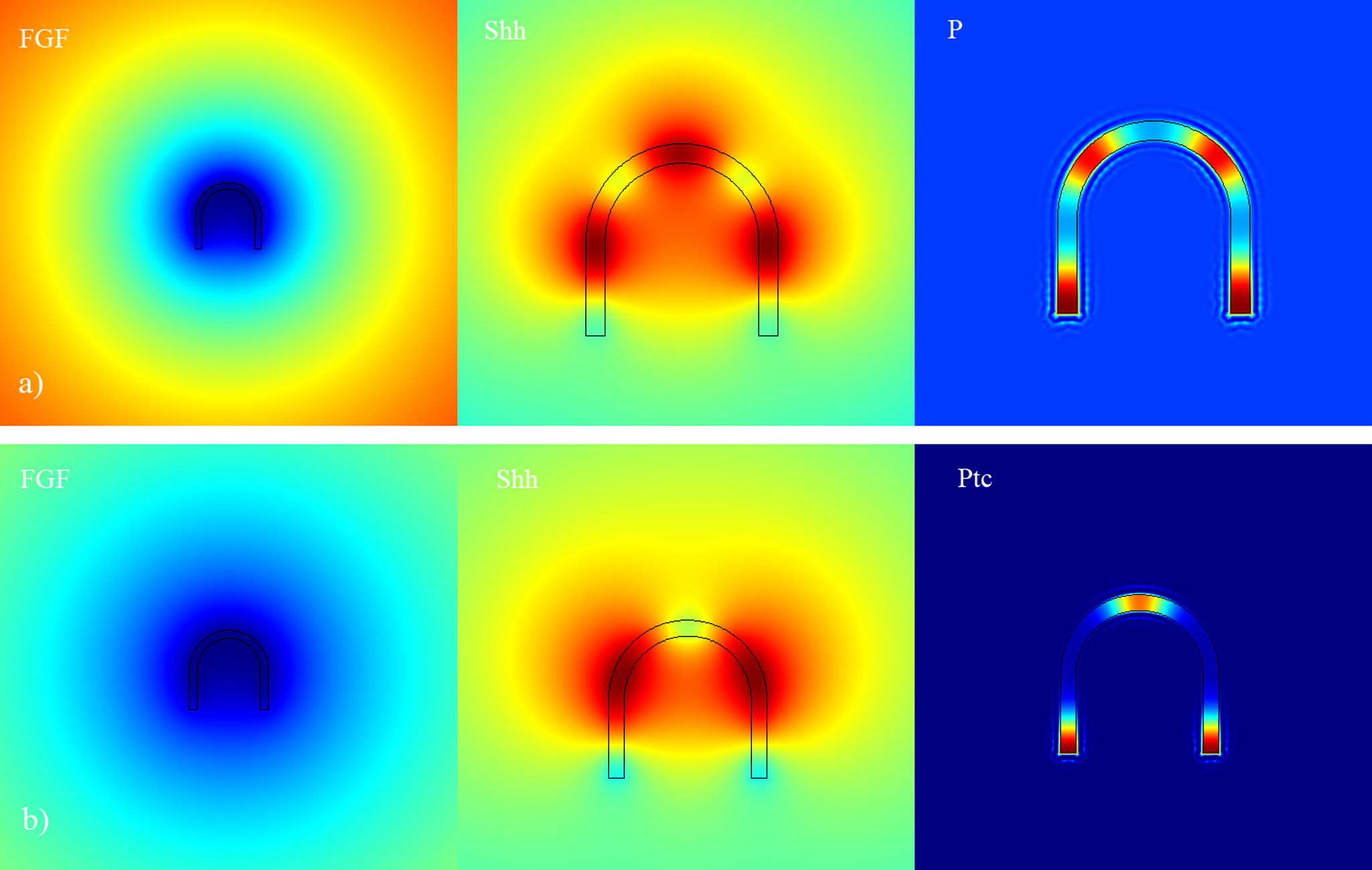

Supplement: Figure S12 — The steady state distributions of FGF10, SHH and receptor Ptc concentrations in a mesenchyme free lung in an a gel. Panels a) and b) show patterns at different FGF10 concentrations. (TIF) [file pcbi.1002377.s012.tif]
